# P56 - Control of allergic inflammation and erythrocytes magnesium level in children with bronchial asthma

**DOI:** 10.1186/2045-7022-4-S1-P111

**Published:** 2014-02-28

**Authors:** Ivan Shishimorov, Alex Perminov, Vitaly Gorbunov

**Affiliations:** 1 Clinical Pharmacology and Intensive Therapy Department, Volgograd State Medical University, Volgograd, Russia

## Purpose

To examine the relationship of severity and duration of allergic inflammation to the erythrocytes magnesium level in children with bronchial asthma (BA).

## Materials and methods

40 patients with a diagnosis of mild to moderate severity were divided into 2 groups: Group 1 included 20 patients experienced disease less than 5 years; Group 2 included 20 patients with disease duration of more than 5 years. All patients received standard treatment of inhaled corticosteroids. Patients were to determine the level of NO in exhaled air using a device NObreath and the concentration of markers - sICAM-1, IL-4, IL-8, IFN-gamma, and magnesium content in erythrocytes. To assess the level of asthma control were used ACT test.

## Results

Patients comparison groups had comparable levels of clinical control of asthma by AST-test. In determining the level of nitric oxide in exhaled air were obtained pre-credibility of the differences between the groups. A more pronounced level of allergic inflammation in patients 2 groups confirmed higher concentrations of its laboratory markers. In determining the level of magnesium in red blood cells have lower it with the content of the group 2 patients.

## Conclusions

The study revealed the discrepancy between clinical asthma control level and laboratory markers of allergic inflammation. In patients with longer experience of the disease on a background of basic therapy revealed higher levels of allergic inflammation and lower levels of magnesium in erythrocytes. Pharmacological correction of magnesium levels may be useful to improve the efficiency of basic therapy of asthma and achieve control of allergic inflammation.

